# UV-induced DNA damage in *Cyclops abyssorum tatricus* populations from clear and turbid alpine lakes

**DOI:** 10.1093/plankt/fbt109

**Published:** 2013-11-11

**Authors:** Barbara Tartarotti, Nadine Saul, Shumon Chakrabarti, Florian Trattner, Christian E. W. Steinberg, Ruben Sommaruga

**Affiliations:** 1 Laboratory of Aquatic Photobiology and Plankton Ecology, Institute of Ecology, University of Innsbruck, Technikerstraße 25, 6020 Innsbruck, Austria; 2 Laboratory of Freshwater and Stress Ecology, Department of Biology, Humboldt-Universität Zu Berlin, Arboretum, Späthstraße 80/81, 12437 Berlin, Germany

**Keywords:** copepods, UV radiation, comet assay, photoprotection, mycosporine-like amino acids, antioxidants

## Abstract

Zooplankton from clear alpine lakes thrive under high levels of solar UV radiation (UVR), but in glacially turbid ones they are more protected from this damaging radiation. Here, we present results from experiments done with *Cyclops abyssorum tatricus* to assess UV-induced DNA damage and repair processes using the comet assay. Copepods were collected from three alpine lakes of differing UV transparency ranging from clear to glacially turbid, and exposed to artificial UVR. In addition, photoprotection levels [mycosporine-like amino acids (MAAs) and lipophilic antioxidant capacity] were estimated in the test populations. Similar UV-induced DNA damage levels were observed among the copepods from all lakes, but background DNA damage (time zero and dark controls) was lowest in the copepods from the glacially turbid lake, resulting in a higher relative DNA damage accumulation. Most DNA strand breaks were repaired after recovery in the dark. Low MAA concentrations were found in the copepods from the glacially turbid lake, while the highest levels were observed in the population from the most UV transparent lake. However, the highest lipophilic antioxidant capacities were measured in the copepods from the lake with intermediate UV transparency. Photoprotection and the ability to repair DNA damage, and consequently reducing UV-induced damage, are part of the response mechanisms in zooplankton to changes in water transparency caused by glacier retreat.

## INTRODUCTION

Organisms from clear alpine lakes have to cope with different stresses including periods of high UV radiation (UVR, 280–400 nm) intensities (Sommaruga, 2001). Many of these alpine (i.e. located above the treeline) lakes located in the Alps are shallow (<15 m depth) and highly transparent to UVR (Laurion *et al*., 2000), while others are glacially turbid (“glacial flour”; inorganic suspended solids) and characterized by low water transparency. In clear alpine lakes in this region, planktonic organisms are exposed to UVR throughout the entire water column, even to the shortest wavelengths in the UVB range (280–315 nm). Zooplankton from these ecosystems, however, have adapted to these environmental conditions by evolving several defense strategies against UVR. For example, the late copepodid and adult life stages of the copepod *Cyclops abyssorum tatricus* (Kozminski), a widespread and common zooplankton species of many high mountain lakes from the Alps, avoid the upper meters of the water column during the day (Tartarotti *et al*., 1999). Levels of carotenoids, which act against photo-oxidative stress, are comparable in *C. abyssorum tatricus* (Tartarotti *et al*., 1999) to those found in other copepod species from diverse alpine aquatic systems (Hessen and Sørensen, 1990; Persaud *et al*., 2007), and the content of UV-absorbing mycosporine-like amino acids (MAAs) in these populations is among the highest reported for freshwater organisms (Tartarotti *et al*., 2004; Tartarotti and Sommaruga, 2006; Persaud *et al*., 2007). In contrast to more sensitive zooplankton taxa such as the cladoceran *Daphnia* (Williamson *et al*., 2001), which may avoid UV by vertical migration (Leech and Williamson, 2001), *C. abyssorum tatricus* from clear alpine lakes is highly resistant to UVR as confirmed by *in situ* experiments assessing its mortality (Tartarotti *et al*., 1999). Copepod populations living in turbid alpine lakes resulting from direct glacier discharge, however, are protected from high levels of UVR. This difference in UV transparency is also reflected at the level of photoprotective responses such as copepod MAA contents (F. Trattner *et al*., in preparation). Apart from MAAs and carotenoids, a variety of antioxidant mechanisms exist. Since DNA damage can also occur via oxidation (Cooke *et al*., 2003), antioxidant levels are important for the organism. Overall, our observations suggest that *C. abyssorum tatricus* populations from alpine lakes of different UV transparency will also respond differently to UV stress at the molecular level.

Regardless of the protective measures, UV exposure causes the formation of damaging photo-products [mainly cyclobutane pyrimidine dimers (CPDs) and pyrimidine (6–4) pyrimidone photo-products ((6–4)PDs)] in the DNA (Mitchell and Karentz, 1993). Zooplankton can, however, repair UV-induced DNA damage to some extent by two processes, nucleotide excision repair (NER) and photo-enzymatic repair (PER). The energetically costly NER is found in almost all taxa without being specific to UV-induced DNA damage (Mitchell and Karentz, 1993), while the less costly PER uses the enzyme photolyase in the presence of longer wavelength UV-A (320–400 nm) and photosynthetically active radiation (PAR, 400–700 nm), reversing pyrimidine dimers (Sutherland, 1981; Mitchell and Karentz, 1993). Photo-enzymatic repair is specific to UV-induced DNA damage, yet it is not present in all taxa (Sancar, 1994). The presence of these repair mechanisms in zooplankton following UV exposure has been shown in a number of studies revealing differences among taxa, species and even life stages (Siebeck and Böhm, 1991; Zagarese *et al*., 1997; Grad *et al*., 2001, 2003; Gonçalves *et al*., 2002; Rocco *et al*., 2002; MacFadyen *et al*., 2004; Ramos-Jiliberto *et al*., 2004; Connelly *et al*., 2009). Light-induced repair is assumed to rely on PER of DNA damage, because survival of UV-stressed *Daphnia* increases in the presence of photo-repair radiation [i.e. longer wavelength UV-A (320–400 nm) and photosynthetically active radiation (PAR, 400–700 nm)] (Siebeck and Böhm, 1991; Grad *et al*., 2001; Williamson *et al*., 2001, 2002; Huebner *et al*., 2006). Molecular evidence of enzymatic photo-repair in *Daphnia* was shown in a study by MacFadyen *et al*. (MacFadyen *et al*., 2004), while other zooplankton species such as the rotifer *Asplanchna girodi* show little to no PER and seem to depend mostly on NER (Sawada and Enesco, 1984; Grad *et al*., 2001, 2003). The significance of DNA repair processes in copepods is not well understood. Photo-repair accounts for the relatively high UV tolerance in some copepod species (Zagarese *et al*., 1997; Gonçalves *et al*., 2002; Williamson *et al*., 2002), whereas little evidence of PER is found in others (Zagarese *et al*., 1997; Tartarotti *et al*., 2000).

Most studies on UV-induced DNA damage and repair in zooplankton so far focus on survival rates after exposure to different UVR/PAR treatments (with and without photo-repair radiation) and dark conditions, while only few show direct evidence of molecular responses by, for example, quantifying cytotoxic photo-products of DNA damage (Malloy *et al*., 1997; MacFadyen *et al*., 2004; Connelly *et al*., 2009). One method for measuring DNA damage in aquatic organisms is the use of single-cell gel electrophoresis or comet assay (see Lee and Steinert, 2003 and Frenzilli *et al*., 2009 for reviews). This method has the advantage that DNA strand breaks are determined in individual cells, only a relatively small number of cells is needed to carry out the assay, the assay can be performed on virtually any eukaryotic cell type, and it is a very sensitive method for detecting DNA damage (see Lee and Steinert, 2003 for a review).

The objective of the current study was to address the question of how copepod populations with different levels of UV protection (i.e. MAAs and lipophilic antioxidant capacity) respond to the effects of UVR at a molecular level, more specifically on the protection dependence of UV-induced DNA damage and repair. We report the results of laboratory experiments aimed to understand the relationship between molecular response mechanisms and environmental changes such as in UV transparency resulting from glacier retreat.

## METHOD

### Sampling

Between July and September 2011, the cyclopoid copepod *C. abyssorum tatricus* Kozminski (Einsle, 1993) was collected from three alpine lakes of differing transparency, ranging from highly UV transparent (Faselfadsee 4; FAS4), UV transparent (Mutterbergersee; MUT) to glacially turbid (Faselfadsee 3; FAS3) (see Table I for lake description). Animals were collected by taking several vertical net (50-μm mesh size) tows made at the center of the lakes in the morning. Upon return to the laboratory, the copepods were maintained at ambient lake water temperature (6–8°C) conditions and exposed to UVR.

**Table I.** Day of sampling, geographic location, altitude, maximum lake depth, lake area, mean conductivity (Cond), mean pH, water optical properties [mean dissolved organic carbon content (DOC), mean turbidity, diffuse attenuation coefficient at 320 nm (*K*_d320_), depth of 1% of surface irradiance for 320 nm UV (Z_1%_) and PAR (Z_1%PAR_), and fraction of the water column to which 1% of the surface irradiance at 320 nm penetrated (*Z*_1%_:*Z*_max_)] on the day of sampling

Lake	Faselfadsee 4 (FAS4)	Mutterbergersee (MUT)	Faselfadsee 3 (FAS3)
Day of sampling	5 July 2011	21 September 2011	29 August 2011
Latitude/longitude	47°04′27″N; 10°13′34″E	47°0′58″N; 11°7′41″E	47°04′15″N; 10°13′15″E
Altitude (m a.s.l.)	2416	2483	2414
*Z*_max_ (m)	15.0	8.1	17.0
Lake area (ha)	1.9	3.8	2.1
Cond (µS cm^−1^)	50.6	5.3	43.2
pH	7.4	6.2	8.0
DOC (mg L^−1^)	0.30	0.74	0.27
Turbidity (NTU)	0.2	0.7^a^	8.6
*K*_d320_ (m^−1^)	0.22	0.82	3.86
*Z*_1%_ (m)	21.27	5.62	1.19
*Z*_1%PAR_ (m)	41.57	12.63	3.68
*Z*_1%_:*Z*_max_	1.42	0.69	0.07

^a^Data from September 2010.

### Set-up for UV exposure experiments

Before the beginning of the experiments, one group of copepods (∼100 copepods per sample; 3–5 replicates) was assayed for DNA damage to establish background levels of the different populations (hereafter *t*_0_). For the exposure experiments, copepods were sorted into Petri dishes (∼100 copepods per dish; 3–5 replicates per treatment) filled with 30 mL of filtered (10 μm mesh) lake water. The copepods were exposed to UVR in the presence of photo-reactivating radiation (four A-340 Q-Panel lamps, Q-Panel and two F36W/860 white daylight lamps, General Electric Lightning). The lamps were placed 25 cm above the dishes and the integration of irradiance values between 280 and 320 nm was 1.4 W m^−2^ (84 J m^−2^ min^−1^). Irradiance measurements were made with an USB4000 UV-VIS fiber optic spectrometer calibrated at Dr M. Blumthaler's laboratory. The spectrum of the Q-Panel lamps is available in Sommaruga *et al*. (Sommaruga *et al*., 1996). Copepods in the control were kept in the dark (dishes covered with aluminum foil). The experiments were done in a temperature-controlled environmental chamber at 6°C and in all cases they had an exposure period of 4 h. As the results of the first experiment with copepods from Lake FAS4 showed no further increase in UV damage over a longer exposure (i.e. 6 h), we used a shorter UV exposure period (2 h) to see a potential dose response (Lake MUT). The reason why we used only a 4-h exposure for the Lake FAS3 experiment is that there were not enough copepods present in the samples (maximum abundance in the lake: 1.2 individuals L^−1^). Thus, animals from Lake FAS4 were exposed for 4 and 6 h, whereas those from Lake MUT were exposed for 2 and 4 h, and from Lake FAS3 only for 4 h. To allow for dark repair after the end of the exposure (6 h for Lake FAS4, 4 h for Lakes MUT and FAS3), the animals were left to recover in the dark for 24 h (3–5 replicates per treatment). The same time was used for those copepods already placed in the dark (i.e. control). After the end of each exposure period and the dark recovery, copepods were checked for mortality, and the comet assay was run immediately.

### Single-cell gel electrophoresis (comet) assay

The assay was conducted according to a modified version of the procedures described by Singh *et al*. (Singh *et al*., 1988), detecting double strand breaks, single strand breaks and alkali labile lesions (Lee and Steinert, 2003). ∼100 copepods (mostly copepodid CIII and CIV live stages, no egg-carrying females) were used for each assay. Copepods were homogenized with a Potter-Elvehjem glass homogenizer in 1-mL Ringer solution (5.0 mM HEPES, 116.0 mM NaCl, 2.9 mM KCl, 1.8 mM CaCl_2_, pH 7.2), left standing for 5 min to allow heavy materials in the extract to settle, followed by transfer of the supernatant (800 µL) into another microcentrifuge tube. After centrifugation for 5 min at 1000 rpm, the cell pellet was resuspended using 70 µL of 0.65% low-melting point agarose diluted in Kenny's salt solution (0.4 M NaCl, 9 mM KCl, 0.7 mM K2HPO4, 2 mM NaHCO3), added onto a frosted slide [pre-coated with 1% normal melting point agarose diluted in TAE solution (0.04 M Tris–acetate and 1 mM EDTA)] and covered with a cover slip. After gel solidification (∼3 min, on ice), slides were placed in a Coplin jar containing lysis buffer (2.5 M NaCl, 0.1 M EDTA, 0.01 M Tris–HCl, 10% dimethyl sulfoxide, 1% Triton X-100) for ∼14 h (4°C). After lysis, slides were washed three times in MQ water, and left standing in unwinding/electrophoresis buffer (0.2 N NaOH, 1 mM EDTA, pH >13) for 20 min. For DNA strand unwinding, slides were transferred into a horizontal gel electrophoresis unit (EC 340 Maxicell Submarine Gel System, Thermo Scientific) filled with unwinding buffer. Electrophoresis was carried out for 20 min at 25 V and 300 mA. Slides were washed three times in 0.4-M Tris (pH 7.5), dehydrated by a 5-min rinse in cold methanol and air dried. Slides were stained with 60 µL of SYBR Green I Stain (1:10 000 dilution of stock dye in TAE buffer). DNA strand breaks in cells were determined using a Zeiss Axiophot 2 inverted fluorescent microscope (200 × magnification). Cell images were projected onto a high-sensitivity CCD camera. A computerized image analysis system (LUCIA software module COMET, Laboratory Imaging, Prague, Czech Republic) was used to determine the relative tail intensity (% DNA in tail). Percentage tail DNA is considered to be the most useful parameter, as it bears a linear relationship to break frequency (Collins, 2004). Fifty randomly selected cells per slide were analyzed from each sample.

### Mycosporine-like amino acids and antioxidant capacity

Copepods for MAA analyses (∼20 copepodid CIII and 10–15 copepodid CIV life stages per sample; triplicates per life stage) were narcotized with CO_2_-enriched water and then directly placed in microcentrifuge tubes, frozen and stored at –80°C. MAAs were extracted according to the most efficient protocol reported for *C. abyssorum tatricus* (Tartarotti and Sommaruga, 2002), with some modifications. Briefly, samples were extracted in 400 µL of 25% aqueous methanol [v/v; MeOH; high-performance liquid chromatography (HPLC) grade) at 45°C for 2 h and frozen at −80°C. Samples were sonicated (30 s continuously at 40 W) on ice at the beginning of the extraction. Prior to HPLC analysis, the samples were cleared by centrifugation (16 000 *g* for 20 min at 4°C) and 80 μL aliquots were injected in a Phenosphere 5-mm pore-size RP-8 column (4.6-mm inner diameter × 25 cm, Phenomenex) protected with a Phenomenex guard column. Samples were run with a mobile phase of 0.1% acetic acid in 25% aqueous MeOH (v/v) and a flow rate of 0.75 mL min^−1^. Peak absorbance measurements were done at 310, 320, 334 and 360 nm in a Dionex system equipped with a diode-array detector (scanning from 200 to 595 nm). Individual peaks were identified by their relative retention time (order of appearance), absorption spectra and by co-chromatography with standards extracted from the marine alga *Porphyra yezoensis*. The total content of specific MAAs in each sample was calculated from HPLC peak areas, using published molar extinction coefficients (see Tartarotti *et al.*, 2001). Concentrations of the different MAAs were normalized to the dry weight of the copepods [expressed as µg (µg dry weight)]^−1^.

For antioxidant capacity measurements, narcotized copepods (∼60 copepodid CIII/CIV life stages per sample; triplicates) were placed in microcentrifuge tubes, frozen and stored at –80°C. The animals were cleaved with added glass beads in a Speedmill (Analytik Jena, Germany) and centrifuged (12 000 *g*, 4 min) using sodium hydrogen phosphate (0.1 M, pH 6.5) as buffer. The cooled supernatant was processed to extract lipid-soluble antioxidants (e.g. tocopherol, vitamins A, D, β-carotene, steroids and aromatic substances) according to Bligh and Dyer (Bligh and Dyer, 1959). The antioxidant capacity was analyzed via photo-chemiluminescence in a PhotoChem device (Analytik Jena, Jena, Germany) based on Popov and Lewin (Popov and Lewin, 1999). In this analytical process, superoxide anion radicals are generated by a photosensitized reaction and detected by luminescence. In biological samples, the radicals may be quenched by antioxidant substances and enzymes. This quenching gives an inverse measure of the antioxidant capacity and is calculated in comparison with a standard substance, such as Trolox for the lipophilic antioxidant capacity. The lipophilic antioxidant capacities were related to the protein content of the copepods (measured according to Bradford, 1976), and expressed as nmol Trolox [mg protein]^−1^_._ Unfortunately, the copepod samples collected from Lake FAS3 on 29 August were lost, thus antioxidant capacity data from 5 July 2011 were used instead.

### UV attenuation and turbidity measurements

The downwelling irradiance was measured with a PUV-501B profiler radiometer (Biospherical Instruments Inc.) at 305, 320, 340 and 380 nm (full bandwidth at half maximum is 8–10 nm), and in the PAR band. Profiles were made at the center of the lakes between 11:00 and 14:00 h local time. The diffuse attenuation coefficient (*K*_d_) in the water column was determined from the slope of the linear regression of the natural logarithm of downwelling irradiance (*E*_d_) versus depth. For turbidity measurements, water samples were collected with a modified Schindler Patalas sampler (5 l) at the center of the lakes from surface to maximum depths (1–2-m depth intervals) and measured with a Turb 430 T turbidimeter (WTW GmbH, Weilheim, Germany).

### Data treatment

Data are reported as mean ± standard deviation, level of significance was set to *P* < 0.05. The significance of differences between unexposed (*t*_0_ and dark controls) and UV-exposed copepods, or MAA concentrations of the different copepod populations was evaluated by one-way analysis of variance (ANOVA).

## RESULTS

### UV exposure experiments

**Fig. 1.**
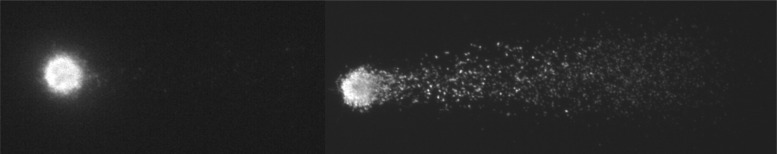
Representative images of DNA damage in *Cyclops abyssorum tatricus* cells (200 × magnification). Left, undamaged cell (background at *t*_0_); right, damaged cell (exposed to UVR plus photo-reactivation radiation for 4 h).

**Fig. 2.**
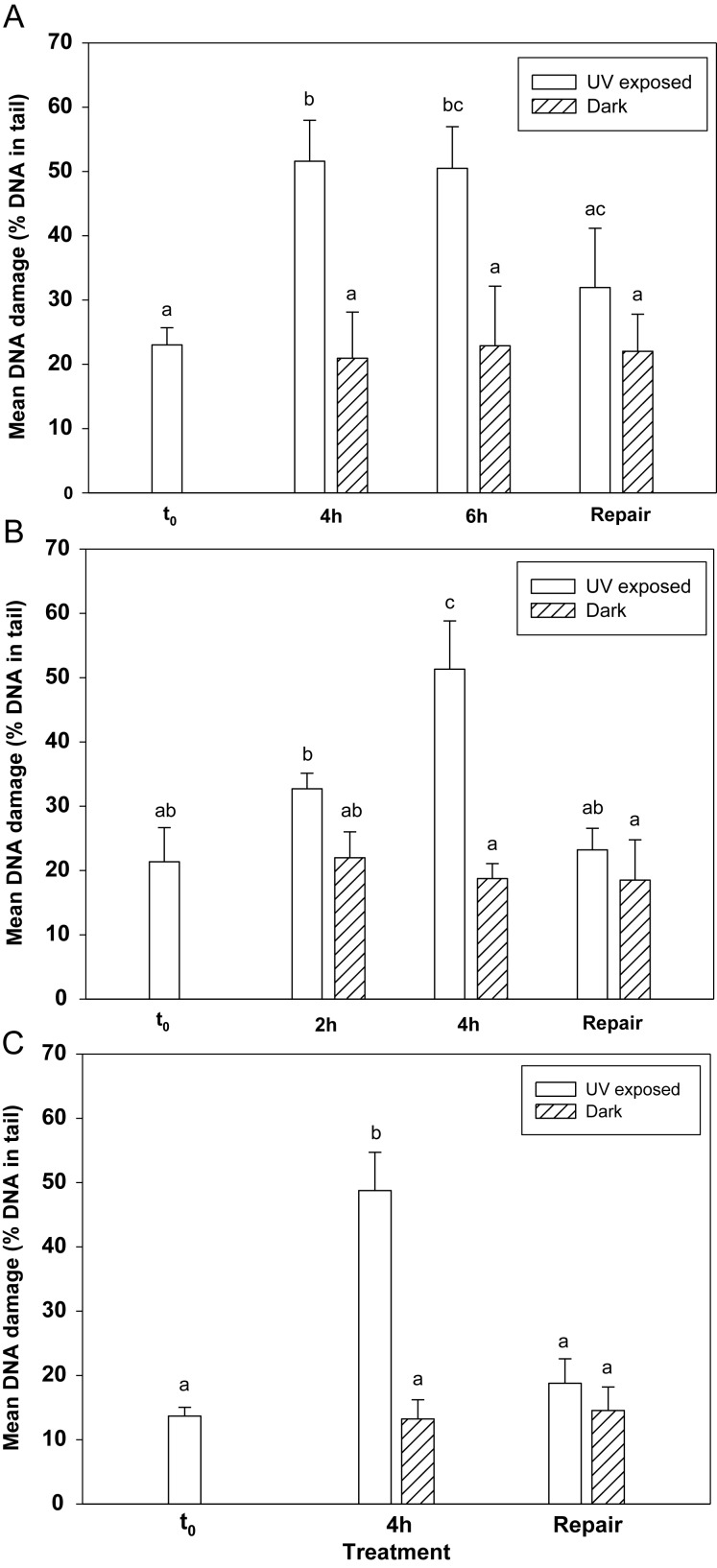
UV-induced DNA damage and recovery in *C. abyssorum tatricus* populations. DNA damage at the beginning of the experiment (*t*_0_), following UV exposure with photo-reactivation radiation (UV exposed), when kept in the dark (dark), and after recovery in the dark for 24 h (repair). (**A**) Faselfadsee 4 (FAS4), (**B**) Mutterbergersee (MUT) and (**C**) Faselfadsee 3 (FAS3). Data are presented as mean % DNA in tail ± standard deviation (*n* = 3–5). Different letters above the bars indicate a significant difference found with ANOVA, all pairwise multiple comparison procedures (Holm-Sidak method, *P* < 0.05) after arcsin square root transformation of the data.

No mortality was observed in the copepods after exposure to UVR in all experiments. Background DNA damage levels at *t*_0_ ranged from 14 to 23% mean DNA in tail, which is within the normal range for environmental studies (Tice *et al*., 2000), whereas up to 52% mean DNA in tail was found in UV-exposed copepods (Fig. 1). Among all three lake populations, the DNA damage levels were similar in the UV-exposed treatments (∼50%), but the background damage (*t*_0_ and dark controls) was lowest in the copepods from Lake FAS3 (Fig. 2). In the copepods from Lake FAS4, significantly higher DNA damage was found after 4 h of UV exposure when compared with the background damage (*t*_0_ and dark control) (Fig. 2A). No further increase in DNA damage was observed after a longer exposure period (i.e. 6 h). DNA strand breaks in the dark controls were not statistically significantly different from the values obtained at the beginning of the experiment (*t*_0_). DNA damage was significantly reduced after post-exposure recovery in the dark when compared with the UV-exposed treatment (Fig. 2A). In the copepods from Lake MUT, DNA damage increased with increasing exposure time (Fig. 2B). Compared with the copepods from *t*_0_ and the dark control, DNA damage was ∼1.5 and ∼2.4 times higher in the animals exposed for 2 and 4 h, respectively. DNA strand breaks were also significantly higher after 4 h of exposure when compared with 2 h (Fig. 2B). Significant decreases in DNA strand breaks after the 24-h post-exposure in the dark were observed (Fig. 2B). In Lake FAS3, DNA damage was significantly higher after 4 h of exposure when compared with the background damage (*t*_0_ and dark control) (Fig. 2C). Similarly as for the other lake populations, DNA damage decreased significantly after recovery in the dark (Fig. 2C).

### Photoprotective compounds

**Fig. 3.**
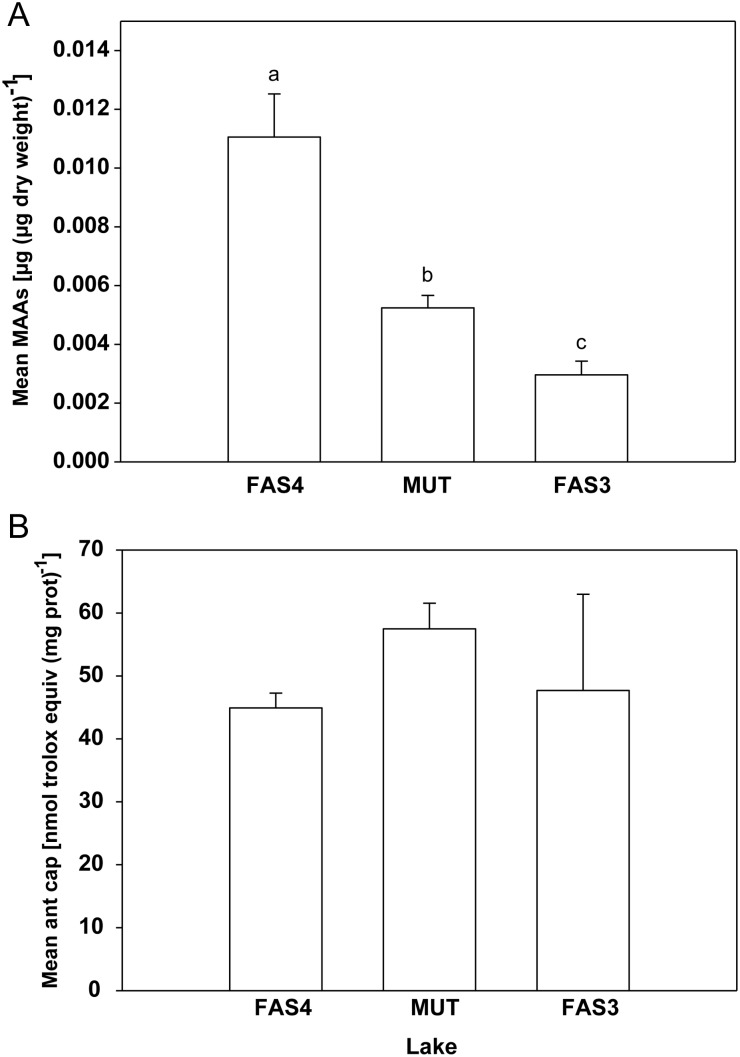
UV protection in *C. abyssorum tatricus* populations. (**A**) Total mean MAA concentrations and (**B**) mean lipophilic antioxidant capacity (ant cap) in *C. abyssorum tatricus* populations from Faselfadsee 4 (FAS4), Mutterbergersee (MUT) and Faselfadsee 3 (FAS3). Error bars indicate standard deviation (*n* = 3–6). (A) Different letters above the bars indicate a significant difference found with ANOVA, all pairwise multiple comparison procedures (Holm-Sidak method, *P* < 0.05).

Up to six MAAs were detected in the copepod samples, with shinorine being predominant (64.4, 37.4 and 88.8% of the total MAA concentration in copepods from Lakes FAS4, MUT and FAS3, respectively). Concentrations of MAAs varied among populations and the highest values were found in the copepods from the Lake FAS4 (Fig. 3A). Copepods from both FAS4 and MUT lakes had significantly higher MAA contents than the ones from FAS3. As for MAA concentrations, the antioxidant capacity (lipid-soluble antioxidants) varied between populations. The highest concentrations were measured in the copepods from MUT (Fig. 3B).

## DISCUSSION

The copepod *C. abyssorum tatricus* commonly occurs in alpine lakes ranging from highly UV transparent to glacially turbid. As expected, UVR caused DNA damage to the copepods; however, the relative extent of damage varied among lake populations living under differing UV stress conditions (Fig. 2). While similar DNA damage levels (∼50% mean DNA in tail) were observed among all three lake populations (Fig. 2), DNA damage at *t*_0_ and in the dark controls was lowest in copepods from the glacially turbid lake (FAS3; Fig. 2C). Although the same copepod species and the same methodological procedures were used for the three populations, the copepods from Lake FAS3 originate from an environment with less UV stress, which may explain the variation in background levels observed among populations. In addition, differences in the sampling date and thus, in sun angle and potential UV pre-exposure levels of the copepods may have influenced background DNA damage levels. Albeit the copepods used in the experiments originated from the whole water column, because samples were taken by vertical net tows, their vertical distribution has an influence on the prior UV exposure levels. Even if the copepods from Lake FAS4 stay close to the lake bottom during the day and the majority of the Lake MUT copepods can also be found in the deepest water layers (unpubl. results), still they are exposed to relatively high UV levels because of the high water transparency in these lakes (Table I). Conversely, the copepods from turbid Lake FAS3, which were more evenly distributed over the whole water column (unpubl. results), were not exposed to UVR (320 nm) below a depth of 1.2 m (Table I).

The high MAA contents and/or high antioxidant capacities of the copepods from the UV-transparent lakes (Fig. 3) may account in part for the relatively lower DNA damage compared with that found in the copepods from the glacially turbid lake. The very high levels of MAAs in the copepods from Lake FAS4 may provide ample UV protection, whereas the animals from Lake MUT had lower MAA contents albeit higher antioxidant capacity, which includes lipid-soluble substances such as carotenoids. That high carotenoid levels do not necessarily imply high MAA contents has been observed in calanoid copepods from North American lakes (Persaud *et al*., 2007) and from high altitude Himalayan lakes (Sommaruga, 2010). Recent studies suggest that MAAs and carotenoids can be complementary photoprotective compounds in copepods, i.e. one is high when the other is low (Hylander *et al*., 2009). Thus, in terms of UV protection, Lake MUT copepods may compensate for their lower MAA content by higher antioxidant capacity, while the copepods from turbid Lake FAS3 showed low overall UV protection. The photoprotective role of antioxidants such as carotenoids in the reduction of DNA damage was suggested by Kim *et al*. (Kim *et al*., 2000). They found that while extensive DNA strand breaks, measured by the comet assay, are observed in late-stage embryos of the grass shrimp (*Palaemonetes pugio*) having only trace amounts of carotenoids, high levels of these protective pigments provide efficient protection from solar damage in early-stage embryos. In addition to the photoprotective compounds we measured, biochemical macromolecules such as proteins and other biomolecules having chromophores that absorb radiation in the UV-B range (Häder and Tevini, 1987) may help the copepods to protect their DNA.

DNA strand breaks in the copepods from Lake MUT were significantly higher (∼1.6-fold) after 4 h of UV exposure than at 2 h (Fig. 2B). This agrees with other studies where clear dose responses in UV-induced DNA damage measured by the comet assay are, for example, observed in late-stage embryos of the crustacean *P. pugio* (Kim *et al*., 2000). Dose-dependent DNA damage induction measured by CPD frequencies is also found in *Daphnia* after exposure to UVR (Connelly *et al*., 2009). Interestingly, in copepods from Lake FAS4, longer exposure times (6 h compared with 4 h) did not result in further DNA damage (Fig. 2A). These results may imply that repair processes such as PER were effective under the longer exposure period, thus preventing the cells from further damage, or that the limit of detection of DNA damage was reached. The range of detection is also limited by the structural organization of the DNA, as strand breaks reach a saturation when all DNA loops are relaxed (see Collins, 2009 for a review). Consistent with the results of our study, a moderate increase in DNA fragmentation (comet assay) at low hydrogen peroxide (H_2_O_2_) concentration with no further increase at higher concentrations was observed in *Oncorhynchus mykiss* spermatozoa (Dietrich *et al*., 2005). The mean maximum DNA damage we observed (up to 52% DNA in tail, Fig. 2) was higher than the values reported in other studies using UVR (Pruski *et al*., 2009) or H_2_O_2_ (Dietrich *et al*., 2005) as stressors and percentage tail DNA as parameter for measuring comets. Dietrich *et al*. (Dietrich *et al*., 2005), however, found up to ∼70% tail DNA in rainbow trout spermatozoa when irradiated with highly damaging short wavelength UV-C radiation.

The ability to repair DNA damage is vital to the organism. The majority of DNA strand breaks in all populations of *C. abyssorum tatricus* was repaired within the recovery period in the dark (Fig. 2). This agrees with studies in the sea anemone *Aiptasia pallida* where nucleotide excision repair, as measured by the comet assay, is initiated during the first 2 h of recovery in the dark, and most strand breaks are repaired within 8 h (Hudson and Ferrier, 2008). Significant damage repair has been also observed in two fish species, *Colossoma macropomum* and *Arapaima gigas* (Groff *et al*., 2010). For both fish species, however, high DNA damage levels were found 12 h post-UV exposure, while significant damage reduction was shown 24 h post-exposure, and a longer recovery period (48 h) resulted in no further reduction of DNA damage. These differences in recovery periods demonstrate how diverse the response mechanisms among taxa are. Typically, an initial rapid phase of DNA repair is followed by slower repair of the remaining damage, and most cell types rejoin single strand breaks (induced, for example, by ionizing radiation or H_2_O_2_) rapidly (Wong *et al*., 2005). Fifty percent of damaged DNA, for instance, is repaired in human white blood cells within 30 min of ionizing radiation exposure, while the remaining strand breaks are only slowly repaired, leaving 5% of the damaged DNA after 24 h post-exposure (Banáth *et al*., 1998). With a similar time course, UV-induced lesions are also repaired by nucleotide excision repair (Wong *et al*., 2005). Although most DNA damage in *C. abyssorum tatricus* was repaired within 24 h, we do not know the onset of the repair processes and whether shorter recovery periods would have been as effective. Apart from dark repair, photo-enzymatic repair mechanisms have been reported for several zooplankton species (Siebeck and Böhm, 1991; Grad *et al*., 2001; Williamson *et al*., 2001, 2002; Huebner *et al*., 2006). In our experiments, we followed the damage response of *C. abyssorum tatricus* to exposure conditions (UVR plus photo-repair radiation) when defense mechanisms such as light repair are available. MacFadyen *et al*. (MacFadyen *et al*., 2004) separated between exposure to UV-B in the presence or absence of photo-reactivating radiation to separate out net DNA damage and PER in *Daphnia*. In these organisms, NER (up to 71%) is about twice as effective as PER in repairing both CPDs and (6–4)PDs. Although most DNA strand breaks were repaired in *C. abyssorum tatricus*, 4–10% of the net damage (i.e. total DNA damage minus repair) still remained after recovery in the dark (Fig. 2), which is similar to the net damage levels observed in *Daphnia* (MacFadyen *et al*., 2004) and in other cell types (Banáth *et al*., 1998).

Temperature dependence of CPD repair rates has been reported for Antarctic zooplankton (Malloy *et al*., 1997) and *Daphnia* (MacFadyen *et al*., 2004). In the latter organism, both DNA repair rates and total DNA damage increase at higher temperatures (15 and 25°C); however, net DNA damage was greater at lower temperatures (5°C) because DNA repair rates are even higher at higher temperatures. MacFadyen *et al*. (MacFadyen *et al*., 2004) suggested that photoprotection may be more effective under low temperature and high UV conditions. Such conditions are typically found in alpine lakes as they are mostly UV transparent with cold water temperatures (lake surface temperature maximum <15°C), and they typically have only a small depth refuge for behavioral UV avoidance (Table I). In addition, several studies show that copepods generally invest mainly in photoprotective pigmentation and less in vertical migration when exposed to UVR (Hansson *et al*., 2007; Hylander *et al*., 2009). Moreover, ice breakup is typically close to the peak UV at summer solstice, exposing planktonic organisms to rapid and significant qualitative and quantitative changes in UVR (Sommaruga, 2001). Thus, copepods that are capable of efficiently utilizing both photoprotection and repair, such as the populations from the UV-transparent study lakes, seem to be well protected from UVR also at the molecular level.

In conclusion, our findings show that photoprotection and DNA repair are important mechanisms in zooplankton to cope with changes in water transparency caused by glacier retreat. In this context, melting of glaciers is expected to increase the short-term lake turbidity, but also to increase lake transparency when the input of glacier-melt waters to the lakes is lost as observed in Lake FAS4.

## FUNDING

This work was supported by the Austrian Science Fund (FWF) [T-236-B17 and V233-B17 to B. T., and P24442-B25 to R. S.], the Tyrolean Science Fund (TWF) [UNI-0404/140 to B. T.], the German Research Foundation (DFG) [STE 673-18/1 to N. S. and S. C.] and the University of Innsbruck [B. T.].

## References

[FBT109C1] Banáth J. P., Fushiki M., Olive P. L. (1998). Rejoining of DNA single-and double-strand breaks in human white blood cells exposed to ionizing radiation. Int. J. Radiat. Biol..

[FBT109C2] Bligh E. G., Dyer W. J. (1959). A rapid method of total lipid extraction and purification. Can. J. Biochem. Physiol..

[FBT109C3] Bradford M. M. (1976). A rapid and sensitive method for the quantitation of microgram quantities of protein utilizing the principle of protein-dye binding. Anal. Biochem..

[FBT109C4] Collins A. R. (2004). The comet assay for DNA damage and repair - principles, applications, and limitations. Mol. Biotechnol..

[FBT109C5] Collins A. R. (2009). Investigating oxidative DNA damage and its repair using the comet assay. Mutat. Res..

[FBT109C6] Connelly S. J., Moeller R. E., Sanchez G. (2009). Temperature effects on survival and DNA repair in four freshwater cladoceran *Daphnia* species exposed to UV radiation. Photochem. Photobiol..

[FBT109C7] Cooke M. S., Evans M. D., Dizdaroglu M. (2003). Oxidative DNA damage: mechanisms, mutation, and disease. FASEB J..

[FBT109C8] Dietrich G. J., Szpyrka A., Wojtczak M. (2005). Effects of UV irradiation and hydrogen peroxide on DNA fragmentation, motility and fertilizing ability of rainbow trout (*Oncorhynchus mykiss*) spermatozoa. Theriogenology.

[FBT109C9] Einsle U. (1993). Crustacea: Copepoda: Calanoida und Cyclopoida.

[FBT109C10] Frenzilli G., Nigro M., Lyons B. P. (2009). The Comet assay for the evaluation of genotoxic impact in aquatic environments. Mutat. Res..

[FBT109C11] Gonçalves R. J., Villafañe V. E., Helbling E. W. (2002). Photorepair activity and protective compounds in two freshwater zooplankton species (*Daphnia menucoensis* and *Metacyclops mendocinus*) from Patagonia, Argentina. Photochem. Photobiol. Sci..

[FBT109C12] Grad G., Burnett B. J., Williamson C. E. (2003). UV damage and photoreactivation: timing and age are everything. Photochem. Photobiol..

[FBT109C13] Grad G., Williamson C. E., Karapelou D. M. (2001). Zooplankton survival and reproduction responses to damaging UV radiation: a test of reciprocity and photoenzymatic repair. Limnol. Oceanogr..

[FBT109C14] Groff A. A., da Silva J., Nunes E. A. (2010). UVA/UVB-induced genotoxicity and lesion repair in *Colossoma macropomum* and *Arapaima gigas* Amazonian fish. J. Photochem. Photobiol. B.

[FBT109C15] Häder D.-P., Tevini M. (1987). General Photobiology.

[FBT109C16] Hansson L.-A., Hylander S., Sommaruga R. (2007). Escape from UV threats in zooplankton: a cocktail of behavior and protective pigmentation. Ecology.

[FBT109C17] Hessen D. O., Sørensen K. (1990). Photoprotective pigmentation in alpine zooplankton populations. Aqua Fenn..

[FBT109C18] Hudson C. L., Ferrier M. D. (2008). Assessing ultraviolet radiation-induced DNA damage and repair in field-collected *Aiptasia pallida* using the comet assay. Internat. Coral Reef Sym. Proc..

[FBT109C19] Huebner J. D., Young D. L. W., Loadman N. L. (2006). Age-dependent survival, reproduction and photorepair activity in *Daphnia magna* (Straus, 1820) after exposure to artificial ultraviolet radiation. Photochem. Photobiol..

[FBT109C20] Hylander S., Boeing W. J., Granéli W. (2009). Complementary UV protective compounds in zooplankton. Limnol. Oceanogr..

[FBT109C21] Kim G. B., Lee R. F., Mitchell D. L. (2000). Damage of grass shrimp (*Palaemonetes pugio*) embryo DNA by summer sunlight followed by DNA repair in the dark. Mar. Biol..

[FBT109C22] Laurion I., Ventura M., Catalan J. (2000). Attenuation of ultraviolet radiation in mountain lakes: factors controlling the among- and within-lake variability. Limnol. Oceanogr..

[FBT109C23] Lee R. F., Steinert S. (2003). Use of the single cell gel electrophoresis/comet assay for detecting DNA damage in aquatic (marine and freshwater) animals. Mutat. Res..

[FBT109C24] Leech D. M., Williamson C. E. (2001). In situ exposure to ultraviolet radiation alters the depth distribution of *Daphnia*. Limnol. Oceanogr..

[FBT109C25] MacFadyen E. J., Williamson C. E., Grad G. (2004). Molecular response to climate change: temperature dependence of UV-induced DNA damage and repair in the freshwater crustacean *Daphnia pulicaria*. Global Change Biol..

[FBT109C26] Malloy K. D., Holman M. A., Mitchell D. (1997). Solar UVB-induced DNA damage and photoenzymatic DNA repair in Antarctic zooplankton. Proc. Natl Acad. Sci. USA.

[FBT109C27] Mitchell D. L., Karentz D., Young A. R., Björn L. O., Moan J., Nultsch W. (1993). The induction and repair of DNA photodamage in the environment. Environmental UV Photobiology.

[FBT109C28] Persaud A. D., Moeller R. E., Williamson C. E. (2007). Photoprotective compounds in weakly and strongly pigmented copepods and co-occurring cladocerans. Freshwat. Biol..

[FBT109C29] Popov I., Lewin G. (1999). Photochemiluminescent detection of antiradical activity. VI. Antioxidant characteristics of human blood plasma, low density lipoprotein, serum albumin and amino acids during in vitro oxidation. Luminescence.

[FBT109C30] Pruski A. M., Nahona S., Escandea M.-L. (2009). Ultraviolet radiation induces structural and chromatin damage in Mediterranean sea-urchin spermatozoa. Mutat. Res..

[FBT109C31] Ramos-Jiliberto R., Dauelsberg P., Zúñiga L. R. (2004). Differential tolerance to ultraviolet-B light and photoenzymatic repair in cladocerans in a Chilean lake. Mar. Freshwater Res..

[FBT109C32] Rocco V. E., Oppezzo O., Pizarro R. (2002). Ultraviolet damage and counteracting mechanisms in the freshwater copepod *Boeckella poppei* from the Antarctic Peninsula. Limnol. Oceanogr..

[FBT109C33] Sancar A. (1994). Mechanisms of DNA excision repair. Science.

[FBT109C34] Sawada M., Enesco H. E. (1984). Effects of UV radiation on the lifespan of the rotifer *Asplanchna brightwelli*. Exp. Gerontol..

[FBT109C35] Siebeck O., Böhm U. (1991). UV-B effects on aquatic animals. Ver. Int. Ver. Theoret. Angew. Limnol..

[FBT109C36] Singh N. P., McCoy M. T., Tice R. R. (1988). A simple technique for quantitation of low levels of DNA damage in individual cells. Exp. Cell Res..

[FBT109C37] Sommaruga R. (2001). The role of solar UV radiation in the ecology of alpine lakes. J. Photochem. Photobiol. B.

[FBT109C38] Sommaruga R. (2010). Preferential accumulation of carotenoids rather than of mycosporine-like amino acids in copepods from high altitude Himalayan lakes. Hydrobiologia.

[FBT109C39] Sommaruga R., Oberleiter A., Psenner R. (1996). Effect of UV radiation on the bacterivory of a heterotrophic nanoflagellate. Appl. Environ. Microbiol..

[FBT109C40] Sutherland B. M. (1981). Photoreactivation. Bioscience.

[FBT109C41] Tartarotti B., Baffico G., Temporetti P. (2004). Mycosporine-like amino acids in planktonic organisms living under different UV exposure conditions in Patagonian lakes. J. Plankton Res..

[FBT109C42] Tartarotti B., Cabrera S., Psenner R. (1999). Survivorship of *Cyclops abyssorum tatricus* (Cyclopoida, Copepoda) and *Boeckella gracilipes* (Calanoida, Copepoda) under ambient levels of solar UVB radiation in two high-mountain lakes. J. Plankton Res..

[FBT109C43] Tartarotti B., Cravero W., Zagarese H. E. (2000). Biological weighting function for the mortality of *Boeckella gracilipes* (Copepods, Crustacea) derived from experiments with natural solar radiation. Photochem. Photobiol..

[FBT109C44] Tartarotti B., Laurion I., Sommaruga R. (2001). Large variability in the concentration of mycosporine-like amino acids among zooplankton from lakes located across an altitude gradient. Limnol. Oceanogr..

[FBT109C45] Tartarotti B., Sommaruga R. (2002). The effect of different methanol concentrations and temperatures on the extraction of mycosporine-like amino acids (MAAs) in algae and zooplankton. Arch. Hydrobiol..

[FBT109C46] Tartarotti B., Sommaruga R. (2006). Seasonal and ontogenetic changes of mycosporine-like amino acids in planktonic organisms from an alpine lake. Limnol. Oceanogr..

[FBT109C47] Tice R. R., Agurell E., Anderson D. (2000). Single cell gel/comet assay: guidelines for in vitro and in vivo genetic toxicology testing. Environ. Mol. Mutagen..

[FBT109C48] Williamson C. E., Grad G., de Lange H. J. (2002). Temperature-dependent ultraviolet responses in zooplankton: implications of climate change. Limnol. Oceanogr..

[FBT109C49] Williamson C. E., Neale P. J., Grad G. (2001). Beneficial and detrimental effects of UV on aquatic organisms: implications of spectral variation. Ecol. Appl..

[FBT109C50] Wong V. W. C., Szeto Y. T., Collins A. R. (2005). The comet assay: a biomonitoring tool for nutraceutical research. Curr. Top. Nutraceut. Res..

[FBT109C51] Zagarese H. E., Feldman M., Williamson C. E. (1997). UV-B induced damage and photoreactivation in three species of *Boeckella* (Copepods, Calanoida). J. Plankton Res..

